# Epstein-Barr Virus-Positive Leiomyosarcoma in Immunocompetent Patients

**DOI:** 10.5146/tjpath.2023.01600

**Published:** 2024-01-22

**Authors:** Hassan Al-Tarawneh, Alpaslan Alp, Gokhan Gedikoglu, Kemal Kosemehmetoglu

**Affiliations:** Mutah University, Faculty of Medicine, Al-Karak, Jordan; Department of Pathology, Hacettepe University, Faculty of Medicine, Ankara, Turkey

**Keywords:** EBER, Smooth muscle tumor, EBV, Leiomyosarcoma

## Abstract

***Objective: *** Epstein-Barr Virus–Associated Smooth Muscle Tumor (EBV-SMT) is a rare tumor with a higher rate of occurrence in unusual locations in the setting of immunodeficiency. In this study, we evaluated a cohort of ordinary leiomyosarcomas (LMS) for the presence of EBV and described the clinicopathological features deviating from routinely diagnosed cases of EBV-SMT.

***Material and Methods:*** The sections of tissue microarrays including 93 classical LMS occurring in various locations were hybridized with EBER and stained for LMP1 antibody using the Leica Bond Autostainer. EBV real-time PCR assay was performed in 2 EBER-positive cases.

***Results: *** Among the 93 LMS cases, 2 non-uterine cases (2.2%) were positive for EBER and negative for LMP1, and were referred to as “EBV-positive LMS”. Both were females in their 6th decade without immunosuppression. EBV real-time PCR assay revealed the presence of EBV in one of the cases. Tumors were located in the pancreas and chest wall. Morphologically, tumors were rather myxoid, multinodular, and composed of long fascicles of spindle cells with intermediate- to high-grade features. High mitotic activity and focal necrosis were present, whereas no accompanying lymphocytes were detected. One of the patients developed metastatic disease after 3 years.

***Conclusion:*** EBV-positive LMS occurring in immunocompetent patients has features distinct from classical EBV-SMT seen in immunosuppressed patients.

## INTRODUCTION

Epstein-Barr Virus–Associated Smooth Muscle Tumor (EBV-SMT) is considered a rare tumor that has a high rate of occurrence in unusual locations in the setting of immunodeficiencies, such as congenital immunodeficiency syndromes, particularly in children, and in relation to the acquired immunodeficiency syndrome (AIDS) or organ transplantation in young adults. Characteristically, it consists of short fascicles of immature neoplastic cells showing smooth muscle differentiation, mild nuclear atypia, low mitotic activity, accompanying lymphocytes, and EBV positivity (1–6). Although EBV-SMT is described in detail in immunocompromised individuals, the presence of EBV in classical LMS of immunocompetent patients could not be shown before in a relatively small series (7). In this study, we performed in-situ hybridization (ISH) for Epstein-Barr Virus Encoded RNA (EBER) in a cohort of 93 classical leiomyosarcoma (LMS) cases to detect the incidence of EBV in ordinary LMS and describe the morphological features of EBV-positive LMS with special emphasis on deviating features from EBV-SMT.

## MATERIALS and METHODS

Four blocks of 3-4 mm diameter tissue microarrays composed of 93 classical LMSs of different locations (55 uterine and 38 non-uterine) were investigated for the presence of EBV. ISH for EBER (Leica Bond Ready to Use ISH) and LMP1 (Thermo, CS1+CS2+CS3+CS4 cocktail, ER2 10 min, 1:100) immunohistochemistry were performed on the tissue microarray slides using the Leica Autostainer Bond Max2. Diffuse nuclear staining for EBER and cytoplasmic staining for LMP1 were regarded as positive. For clinicopathological comparison, 7 consequent cases diagnosed as EBV-SMT between 2000 and 2018 were retrieved from the pathology archives, and the relevant clinicopathological findings were recorded. A consult case of a 77-year-old female with multiple masses in the thigh, pancreas, and lung was excluded due to the limited core biopsy taken from the lung, lack of clinicopathological correlation, diffuse CD10, and focal desmin and SMA expression, despite morphological features consistent with EBV-SMT or LMS and patchy EBER positivity.

The deparaffinization process was applied to the specimens of the two EBER-positive LMS cases that contained five sections of 10 mm. Nucleic acid extraction from the specimen was performed by using the m2000 sp automated extraction instrument (Abbott Molecular Inc., USA). After the DNA extraction step, the detection of EBV was performed by using the Abbott Real-Time EBV PCR kit (Abbott Molecular Inc., USA) in the m2000 rt real-time PCR instrument. The amplification target was a highly conserved region of the *BLLF1* gene that encodes the gp350/220 envelope glycoprotein. Internal control was also used to check the overall internal process, including DNA extraction and possible PCR inhibition. The manufacturer’s lower limit of detection was reported as 115 IU/mL.

## RESULTS

Among 93 classical LMS, only 2 cases (2% of all LMS, 5% of all non-uterine LMS) were positive for EBER, both of which were non-uterine cases, with one located within the pancreas, and the other on the chest wall. These patients had no known history of congenital or acquired immunosuppression, autoimmune disease, or steroid use. HIV tests were negative in both patients. White blood cell counts were 6100/mL (28% lymphocytes) and 9600/mL (%32 lymphocytes), respectively. Clinicopathological characteristics are summarized in Table 1. LMP1 was negative in both cases. The EBV real-time PCR assay was positive in one of the cases (case 2).

**Table 1:** Clinicopathological characteristics of the presented cases.

**#**	**Age**	**Sex**	**Location**	**Largest Size (cm)**	**Multifocality**	**Immunodef. State**	**Mitosis (/10 hpf)**	**Pleo**	**Necrosis**	**Ly**	**Gr**	**IHC**	**Associated Clinical Conditions**	**Tx**	**Follow-up (mos)**
1	56	F	Pancreas, spleen	19x8x8	solitary	None	2	Prominent	Present	-	High	SMA+, desmin+, h-caldesmon+	cRCC, pRCC	S, CTx	84, with mets
2	55	F	Chest wall	7x5x5	solitary	None	20	Prominent	Focal	-	High	SMA, desmin, h-caldesmon+	-	S, CTx	13

**cRCC:** Clear cell renal cell carcinoma, **CTx:** Chemotherapy, **F:** Female, **Gr:** Grade, **hpf:** High power field, **IHC:** Immunohistochemistry, **Immunodef:** Immmunohdeficiency, **ly:** Accompanying lymphocytes, **met:** Metastasis, **pleo:** Pleomorphism, **pRCC:** Papillary renal cell carcinoma, **S:** Surgery, **SCC:** Squamous cell carcinoma, **SMA:** Smooth muscle actin, **Tx:** Treatment.

**Figure 1:**
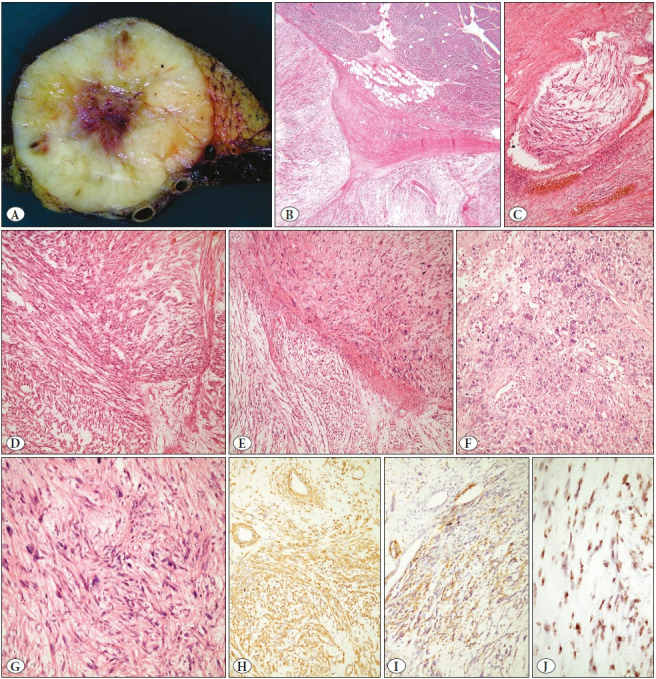
EBV-positive LMS Case #1: **A**) Solid, fleshy, cream-colored tumor with a central stellate scar. Also, note the close relation to the vessels. **B**) Multinodular myxoid spindle cell tumor arising from the pancreas (40xH&E). **C**) Vascular involvement/infiltration (100xH&E). **D**) Intersecting bundles of bland spindle cells with eosinophilic cytoplasm and hyperchromatic nuclei (200xH&E) **E**) Transition to high-grade areas (200xH&E) **F**) High-grade areas with prominent nuclear pleomorphism and hyperchromasia (200xH&E) **G**) High mitotic activity is readily seen (400xH&E). Neoplastic cells were positive for SMA (**H**, 200x), desmin (**I**, 200x) and EBER (**J**, 400x)

**Figure 2:**
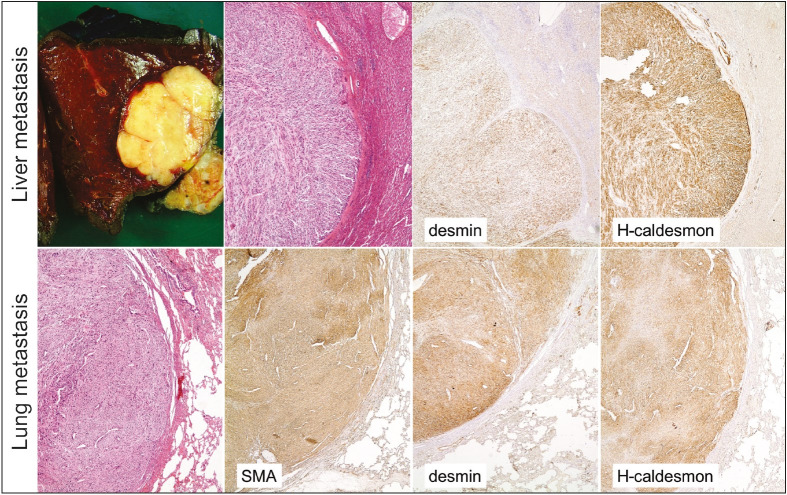
Lung and liver metastases of EBV-positive LMS Case #1 showing similar morphology and immunophenotype.

The first case was a 56-year-old female with a 19x8x8 cm mass at the body and tail of the pancreas. She also had a 5 cm, exophytic right renal mass, diagnosed as conventional renal cell carcinoma following radical nephrectomy performed 2 months after pancreatectomy and splenectomy. On macroscopy, a cream-colored, well-circumscribed, solid, multinodular tumor with a stellate scar at the center was located in the body of the pancreas and was also closely associated with the surrounding vascular structures (Figure 1). Morphologically, the multinodular tumor was composed of long fascicles of spindle cells with myxoid background and intermediate-grade features (Figure 1). Focal areas of higher nuclear grade, epithelioid cells, necrosis, and vascular invasion were present. Mitosis was 1-2/10 HPF. Neoplastic cells were positive for SMA, desmin, CD34, and EMA while S100 and c-kit were negative. She received adjuvant chemotherapy and underwent several metastatectomies of the lung and liver after 3 years and 6 years, respectively. Metastases were consistent with leiomyosarcoma (Figure 2). Two years later, a 2 cm left kidney mass was taken out by partial nephrectomy and diagnosed as type 1 papillary renal cell carcinoma. She was lost to follow up with enlarging pulmonary metastatic nodules after 7 years.

**Figure 3:**
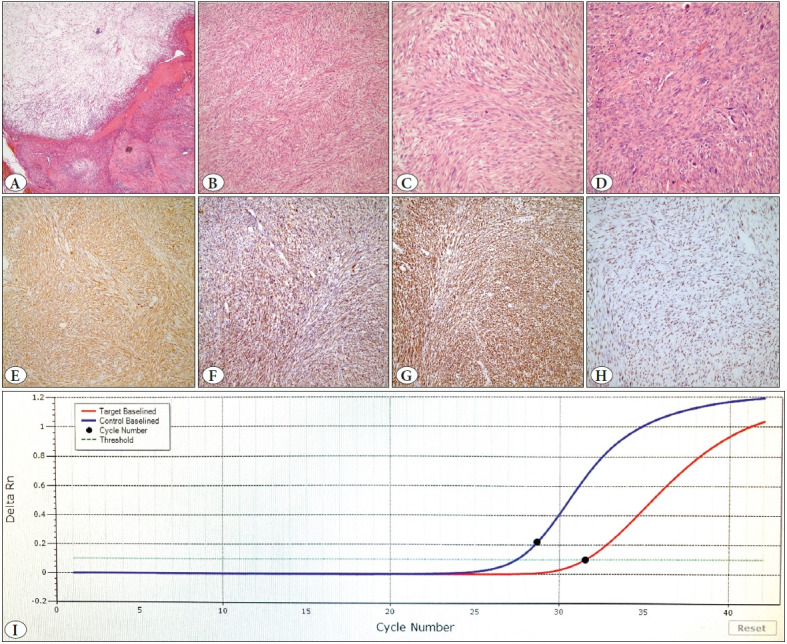
EBV-positive LMS Case #2: **A**) Multinodular tumor composed of prominent myxoid and solid areas with a collagenous background (40x H&E). **B**) Cellular areas resembling fibrosarcoma (100xH&E) **C**) Higher magnification shows spindle cells with relatively bland, cigar-shaped nuclei and contrasting brisk mitotic activity (200xH&E). **D**) Other areas with prominent nuclear pleomorphism (200xH&E). Neoplastic cells diffusely expressed SMA (**E**, 100x), desmin (**F**, 100x), h-caldesmon (**G**, 100x), and EBER (**H**, 100x). **I**) EBV real-time PCR assay was positive with a cycle threshold (Ct) value of 31.54.

The second case was a 55-year-old female with a 7x5x5 cm soft tissue mass located in the chest wall beneath the right breast and infiltrating the right anterior parts of the 9-10th ribs. The patient underwent marginal surgical excision. The tumor was vaguely nodular with myxoid areas and composed of long intersecting fascicules composed of pleomorphic spindle cells with a mitotic activity of 20/10 HPF. Bone and pleural invasion and focal areas of necrosis were also present. Neoplastic cells diffusely expressed SMA, desmin, and h-caldesmon, while pan-keratin (AE1/3) was negative (Figure 3). She received adjuvant chemotherapy and was lost to follow-up after 13 months.

## DISCUSSION

EBV is involved in the pathogenesis of various tumors: 1) carcinomas, either prevalently such as nasopharyngeal carcinoma, or only certain subsets such as gastric carcinomas; 2) a wide range of lymphoproliferative disorders such as Burkitt lymphoma, Hodgkin lymphoma, and nasal type T/NK cell lymphoma to name a few; 3) some rare tumors such as Epstein-Barr virus-associated smooth muscle tumor (EBV-SMT) and inflammatory pseudotumor-like follicular dendritic cell tumor (IMT-FDCT)(8). Among these, EBV-SMT is a relatively uncommon neoplasm associated with immunodeficiency. It has been described in patients infected with human immunodeficiency virus (HIV/AIDS), in the posttransplant setting and those with congenital immunodeficiency. The incidence rate of post-transplant EBV-SMT is reported as 1-2% in kidney transplant patients (3). Various anatomic sites, including the retroperitoneum and soft tissues, can be affected by EBV-SMT with a tendency to multifocal involvement. On morphological grounds, tumors are usually indistinguishable from an ordinary low-grade LMS or leiomyoma; however, the presence of variable numbers of intratumoral lymphocytes and EBER positivity are the unique defining features of this tumor (4,6). Although many EBV-SMT cases have been reported especially after the rise of HIV/AIDS, no studies have investigated the incidence of EBV in common LMS. In our study, 2 of 93 LMS were found positive for EBV and both of them were extra-uterine with an overall incidence of 2.2% of total LMS and 5% of the extra-uterine LMS. Neither of the patients had experienced immunosuppression at the time of presentation. These cases could be classified as either EBV-SMT or “EBV-positive LMS”; however, even with the small number of cases, the term “EBV-positive LMS” sounds better since our 2 cases were high-grade sarcomas with prominent pleomorphism occurring in immunocompetent patients, lacked lymphocytes, and had a poorer prognosis than EBV-SMT (Table 2).

**Table 2:** Clinicopathological comparison between “EBV-SMT” and the proposed term “EBV-positive LMS”.

	**EBV-positive LMS**	**EBV-SMT**
Age	The elderly	Children and young adults
Background	No immunodeficiency	Immunodeficiency
Location	Non-uterine, soft tissue	Mainly visceral, soft tissue
Multifocality	Solitary	Multiple
Morphology	High-grade LMS No lymphocytes	No grading (reminiscent of leiomyoma or low-grade LMS) Some with Immature small cells Accompanying lymphocytes
Outcome	Poor	Depends on underlying condition

Apart from commonly seen *TP53, RB1 *and* PTEN* alterations, genomic and transcriptomic investigations have uncovered three specific subtypes LMS that likely develop from distinct lineages of smooth muscle cells: 1) Dedifferentiated LMS with myogenic differentiation and high immune cell infiltration, 2) Tumor arising in the abdomen or extremities with vascular smooth muscle phenotype, low mutational burden, and a better prognosis, and 3) Tumors primarily of gynecological origin with dystrophin alterations (9,10). One of our patients (case #1) is more likely to correlate with LMS subtype 2, having features such as intraabdominal tumor of probable vascular origin, lack of prominent inflammatory cell infiltration, and a relatively smoldering clinical course. Gynecological LMS has been shown to be molecularly different from soft tissue LMS. Along those lines, we have shown that EBV does not seem to play any role in the etiology of the uterine LMS of immunocompetent patients, as none of the 55 uterine LMS cases was positive for EBER. In the literature, there is only one case report in which EBV was demonstrated in the uterine LMS of a 40-year-old woman one year after bilateral lung transplantation due to sarcoidosis (11). Given the fact that EBV-SMT can occur in many locations such as blood vessels, liver, spleen, colon, and lung in the setting of immunosuppression, it is not surprising to encounter a case in the uterus.

The pathogenesis and latency type of EBV-SMT is not well-known. MYC overexpression and activation of the mTOR/Akt pathway are considered the main events in EBV-derived smooth muscle proliferation (12). There are controversial data on the expression of LMP1 and other EBV-related proteins in EBV-SMT (8). The complete absence of LMP1 in all presented cases suggests that type I latency might be involved in EBV-SMT and EBV-positive LMS. However, previously reported del-LMP1 variant and EBNA2 expressions or a possible LMP2 might also be involved in these tumors, suggesting type II or more likely type III latency similar to post-transplant lymphoproliferative disorders (1,13). The event underlying multifocality (whether metastatic spread or multifocal occurrence) has been addressed by Deyrup et al. by stating that the separate nodules in a given patient are clonally distinct, therefore representing different tumors from multiple/multifocal infectious events rather than metastasis (1).

EBER is recommended before diagnosing any smooth muscle tumors seen in patients who have a history of immunodeficiency (2). Regarding our findings, we also recommend EBER testing in diagnosing smooth muscle tumors of non-uterine locations, regardless of the grade of tumor and immune status of the patient. Although the investigation of EBV in leiomyosarcoma in immunocompetent patients seems to be a non-sense and money consuming practice, it might also have a predictive value in the management of treatment. Rapamycin, an mTOR inhibitor, has been reported to be effective on EBV-positive B-cell lymphomas by inhibiting cell cycle (14,15) and post-transplant EBV-SMT (3).

The differential diagnosis of classical EBV-SMT includes Kaposi sarcoma, mycobacterial spindle cell pseudotumor, and myopericytoma to some extent, as these tumors have a relatively low-grade morphology and are seen in immunosuppressed individuals. However, EBV positive LMS typically presents in immunocompetent patients and shows high-grade morphology. The differential diagnosis is therefore different from EBV-SMT and includes common differentials of LMS, such as GIST, dedifferentiated liposarcoma, pleomorphic sarcoma, malignant peripheral nerve sheath tumor, as well as some other EBV-related conditions, particularly inflammatory pseudotumor-like follicular dendritic cell tumor (IMT-FDCT). IMT-FDCT is regarded as a subgroup of follicular dendritic cell sarcomas and commonly presents as a solitary, indolent, painless mass located at extranodal sites such as the spleen, liver, and tonsils. Histologically, it consists of storiform fascicles of plump spindle cells with vesicular nuclei and accompanying prominent inflammatory cells and expresses SMA along with dendritic markers, such as CD21, CD23, and CD35, but desmin or h-caldesmon are consistently negative. An answer to the prompt question “Are there any other sarcomas positive for EBV?” is partially given by Lenze et al., as there were no EBV-positive cases in 44 synovial sarcomas studied (16). The presence of EBV in other types of sarcomas remains to be elucidated.

In conclusion, we have identified the presence of EBV in a subset of non-uterine LMS in immunocompetent patients, for which we proposed the term “EBV-positive LMS” due to the presence of clinical and pathological features distinct from EBV-SMT classically seen in immunosuppressed patients.

## Conflict of Interest

No conflict of interest.
